# Molecular epidemiology of a carbapenem-resistant *Serratia marcescens* outbreak during the COVID-19 pandemic

**DOI:** 10.3389/fmicb.2025.1525543

**Published:** 2025-07-02

**Authors:** Letícia Fernandes da Rocha, Grazielle Motta Rodrigues, Gabriela Simões de Oliveira, Aymê Duarte Echevarria, Priscila Wink, Fabiana Volpato, Mayana Berdichevski, Larissa Lutz, Dariane Castro Pereira, Afonso Luís Barth, Andreza Francisco Martins

**Affiliations:** ^1^Unidade de Microbiologia e Biologia Molecular, Hospital de Clínicas de Porto Alegre, Porto Alegre, Brazil; ^2^PPGCM - Programa de Pós-Graduação em Ciências Médicas, Universidade Federal do Rio Grande do Sul, Porto Alegre, Brazil; ^3^PPGCF - Programa de Pós-Graduação em Ciências Farmacêuticas, Universidade Federal do Rio Grande do Sul, Porto Alegre, Brazil; ^4^Departamento de Microbiologia, Imunologia e Parasitologia, Instituto de Ciências Básicas, Universidade Federal do Rio Grande do Sul, Porto Alegre, Brazil; ^5^LABRESIS - Laboratório de Pesquisa em Resistência Bacteriana, Hospital de Clínicas de Porto Alegre, Porto Alegre, Brazil; ^6^Departamento de Microbiologia, Imunologia e Parasitologia, Instituto de Ciências Básicas, Universidade Federal do Rio Grande do Sul, Porto Alegre, Brazil

**Keywords:** *Serratia marcescens*, blaKPC, single-locus sequence typing, IncP6 plasmid, fliC

## Abstract

**Introduction:**

*Serratia marcescens* is a significant causative agent of hospital-acquired infections (HAIs), particularly in intensive care units (ICUs). Carbapenem resistance represents a major concern in HAI management, as carbapenem-resistant bacteria can trigger outbreaks in hospital settings. While molecular evaluation of outbreaks typically relies on pulse field gel electrophoresis (PFGE) or core genome multilocus sequence typing (cgMLST) methods, alternative rapid, reliable, and cost-effective methods for assessing clonal relatedness are needed.

**Methods:**

This study aimed to characterize a carbapenem-resistant *S. marcescens* outbreak that occurred during the COVID-19 pandemic in a tertiary care hospital, using the flagellin gene as a single-locus sequence typing (SLST) method. In addition, we evaluated the genetic context of carbapenemase genes through whole-genome sequencing (WGS).

**Results:**

Among the 170 carbapenem-resistant *Serratia marcescens* isolates recovered, high resistance to gentamicin, ciprofloxacin, and cefepime was observed. The predominant carbapenemase gene detected by qPCR-HRM was blaKPC (92.2%). Phylogenetic analysis of the flagellin gene grouped the sequences into two distinct clades, with all outbreak-related *bla*_KPC_-positive *S. marcescens* isolates clustering within clade B. The *bla*_KPC_ gene was carried on an IncP6 plasmid.

**Discussion:**

Our findings indicate that the flagellin gene serves as an effective marker for characterizing carbapenem-resistant *S. marcescens* carrying *bla*_KPC_, confirming that the outbreak was caused by the clonal expansion of isolates harboring *bla*_KPC_ on an IncP6 plasmid.

## 1 Introduction

*Serratia marcescens* is a ubiquitous, fermentative, rod-shaped Gram-negative bacteria belonging to the Enterobacterales order. This organism typically exhibits multiple resistance mechanisms, including intrinsic resistance to polymyxins, which significantly limits therapeutic options (Iguchi et al., 2014). As an opportunistic pathogen, *S. marcescens* has been associated with high mortality rates, particularly among immunocompromised patients, during hospital outbreaks (Šiširak, 2013; Iguchi et al., 2014).

Carbapenems are the primary antibiotics used to treat infections caused by *Enterobacterales*, including strains of *S. marcescens* that are resistant to other antimicrobials (da Silva et al., 2021). However, there has been a significant increase in carbapenem-resistant Enterobacterales (CRE) worldwide, particularly in recent years. This rise has been especially noted during the COVID-19 pandemic period, when an overall increase in CRE incidence was documented (Hamers et al., 2022; Pintado et al., 2022).

The first report of a plasmid-encoded carbapenem-hydrolyzing enzyme (KPC-2) in *S. marcescens* was documented in Hangzhou, China. The three isolates obtained from patients at a hospital in China exhibited identical plasmid profiles, indicating that the same plasmid had been transmitted among these *S. marcescens* isolates (Zhang et al., 2007). Currently, nosocomial infections caused by carbapenem-resistant *Serratia* spp. have become increasingly common worldwide, including in Brazil, and are typically attributed to carbapenemase production (Cayô et al., 2017; Barberino et al., 2018; Streling et al., 2018; NOTA TÉCNICA No. 74/2022-CGLAB/DAEVS/SVS/MS-Agência Nacional de Vigilância Sanitária-Anvisa, 2025).

Prompt and accurate identification of sources and transmission routes is crucial for implementing infection control measures and preventing the further nosocomial spread of bacteria. DNA-based typing methods, such as multi-locus sequence typing (MLST), have been developed for key human pathogens. For *Serratia marcescens*, an established MLST scheme is available on PubMLST, which currently includes 1832 sequence types (STs).^1^ This scheme has proven to be valuable for the molecular characterization of *S. marcescens* strains and serves as an important tool for epidemiological surveillance (Martineau et al., 2018). Currently, whole-genome sequencing (WGS)-based typing is employed for the majority of bacterial species, including *S. marcescens* (Zingg et al., 2017; Muyldermans et al., 2021). However, both MLST and WGS are considered time-consuming, labor-intensive, and expensive methods. In contrast, techniques utilizing single- or double-locus sequence typing have been successfully employed for the rapid assignment of clonal lineages in various bacterial species (Weissman et al., 2012; Pournaras et al., 2014; Fernández-Huerta et al., 2020; Magalhães et al., 2020).

During the 2-year period of the COVID-19 pandemic, we observed an increase in infections caused by carbapenem-resistant *S. marcescens* (outbreak) at our institution, which is a tertiary care hospital in southern Brazil. Therefore, we evaluated a novel approach using the flagellin gene as a single-locus sequence typing (SLST) method for the molecular characterization of *S. marcescens* isolates. In addition, we investigated the genetic environment of the carbapenemase genes present in the outbreak isolates.

## 2 Materials and methods

### 2.1 Isolate collection and identification

The study was conducted at Hospital de Clínicas de Porto Alegre, Porto Alegre, Brazil, which is an 860-bed tertiary care university hospital. During a surveillance study focused on carbapenem-resistant Enterobacterales, a total of 170 *S. marcescens* isolates non-susceptible to meropenem—according to the European Committee on Antimicrobial Susceptibility Testing (EUCAST) criteria, (Eucast: MIC Determination, 2025)—were obtained from January 2020 to January 2022. The incidence rates of meropenem-non-susceptible *S. marcescens* (MNSSm) per 1,000 patient-days were evaluated for each month to monitor the increase in the case numbers.

Only one isolate from each patient was included. The isolates were identified by mass spectrometry using the VITEK^®^ MALDI-TOF MS system (bioMérieux, France) and MYLA^®^ (version 3.0) for clinical use.

### 2.2 Antimicrobial susceptibility profile

Antimicrobial susceptibility was evaluated for all isolates using the disc diffusion method following the EUCAST guidelines (EUCAST, 2024). The antibiotics tested included amikacin, cefepime, ciprofloxacin, norfloxacin, ceftazidime, gentamicin, meropenem, piperacillin/tazobactam, and sulfamethoxazole/trimethoprim. The susceptibility profile of tigecycline was determined through broth microdilution following the EUCAST guidelines (EUCAST, 2024), and quality control of this test was performed in parallel using *E. coli* ATCC 25922.

Minimum inhibitory concentrations (MICs) of meropenem, ceftazidime-avibactam, and meropenem-vaborbactam were determined for a subset of 69 isolates using concentration gradient strips (MTS, Liofilchem, Inc., Waltham, MA) according to the EUCAST guidelines. The isolates were selected based on recovery data (during the outbreak period). One isolate per patient was included, sourced from different care units, with at least one isolate collected each month.

### 2.3 Molecular detection of carbapenemase genes

Total genomic DNA was extracted from the isolates by thermal lysis (Dashti et al., 2009), and DNA concentration and purity were evaluated using a NanoDrop 2000 spectrophotometer (Thermo Scientific, Wilmington, DE, United States), with DNA concentrations ranging from 20 to 50 ng/μL. For the qPCR reactions, 1 μL of DNA template was used. The presence of carbapenemase genes was detected using multiplex high-resolution melting real-time PCR (qPCR-HRM) with primers previously described by Monteiro et al. (2012) for *bla*_IMP_, *bla*_VIM,_ *bla*_NDM-1,_ *bla*_KPC,_ *bla*_GES,_ and *bla*_OXA-48-like_.

### 2.4 Single-locus sequence typing

Reference sequences of the flagellin (*fliC*) gene from *S. marcescens* (Jimenez et al., 2020; Moradigaravand et al., 2016; Iguchi et al., 2014; Nodari et al., 2017; Supplementary Table S1) were extracted, aligned, and trimmed to identify the polymorphic region. A phylogenetic tree was reconstructed to compare the relationship between these sequences, and the best region was selected to design the primers using Geneious 9.0 (Kearse et al., 2012). The primers fliC_F (5′-CGCTTCTCAGTCCCGTATCC-3′) and fliC_R (5′-AATAGCC CGATTCCCCCG-3′) were designed to be complementary to the positions 701–1,150 of the *fliC* gene, resulting in a product length of 450 bp.

Total genomic DNA from the 69 isolates was extracted and evaluated according to the protocol cited above (Dashti et al., 2009). In addition, we also sequenced a meropenem-susceptible isolate using Sanger sequencing to serve as an outgroup in the phylogenetic tree. PCR amplification of the *fliC* gene was carried out using 10 ng of DNA template and Platinum^®^ Taq DNA Polymerase (Invitrogen Corporation, United States). The PCR conditions were as follows: 94°C for 5 min, followed by 35 cycles of 94°C for 30 s, 64°C for 45 s, and 72°C for 30 s, with a final extension at 72°C for 5 min. The amplified products were analyzed using 1.5% agarose gel electrophoresis (40 min at 110 v) and purified using ExoSAP-IT PCR Product Cleanup (Afymetrix, Santa Clara, CA, United States).

For Sanger sequencing, the PCR products were labeled using the BigDye Terminator v3.1 Cycle Sequencing Kit (Applied Biosystems, Foster City, CA, United States) and purified using the BigDye XTerminator Purification Kit (Applied Biosystems, Foster City, California, United States). The samples were sequenced in both forward and reverse directions using the ABI 3500 Genetic Analyzer (Applied Biosystems, Foster City, CA, United States).

Phylogeny was reconstructed using IQTree (Nguyen et al., 2015) from consensus sequences generated by aligning a *fliC* gene fragment with MAFFT v7.475 (Katoh and Standley, 2013), using 20 reference sequences (Supplementary Table S1). This fragment was created by systematically removing nucleotides from both ends to identify a DNA sequence that can resolve all phylogenetic clades, aligning with the previously published phylogeny inference (Jimenez et al., 2020). Subsequently, the sequences of this fragment obtained from the isolates in this study were aligned (using MAFFT v7.475) and subjected to maximum likelihood (ML) analysis under the K80 nucleotide substitution model, as selected by the ModelFinder application (Kalyaanamoorthy et al., 2017). Branch support was assessed using the approximate likelihood-ratio test based on the Shimodaira–Hasegawa procedure (SHaLRT) with 1,000 replicates. The phylogenetic tree was visualized using MEGA X (v.10.2.3) (Kumar et al., 2018).

### 2.5 Sequencing and plasmid characterization

One isolate recovered during the outbreak was sequenced using both Illumina MiSeq (2 × 250 bp; average coverage ∼100×) and MinION (R9.4 flow cell) for plasmid characterization. Genomic DNA was extracted from colonies grown in BHI broth (KASVI^®^) using the QIAamp DNA Mini Extraction Kit (QIAGEN^®^). DNA concentration was determined using the Qubit dsDNA HS Assay Kit with a Qubit 4 fluorometer (Thermo Fisher Scientific), and fragment lengths were assessed using TapeStation 2,200 (Agilent, United Kingdom). The quality of the DNA was determined using NanoDrop™, and the 260/280 ratio was considered.

The paired-end library was constructed using the Nextera XT DNA Library Prep Kit (Illumina), while for long reads (MinION; fast model base-calling; Q ≥8; Guppy v6.3.9; MinKNOW 22.10.10), the library was prepared using the Rapid Barcoding Sequencing Kit (SQK-RBK004; Oxford Nanopore), following the manufacturer’s protocols.

Raw short reads were quality-trimmed (Q > 30) and assembled using CLC Genomics Workbench 23. Antimicrobial resistance genes were identified (contigs >200 bp; >10x average coverage) *in silico* using the QIAGEN Microbial Insight-Antimicrobial Resistance database (QMI-AR). Plasmid replicon typing and IS typing were performed using the PlasmidFinder (2.0.1) and MobileElementFinder (v1.0.3) databases, respectively.

CLC Genomics Workbench (v. 23.0) was used to extract reads from base-called MinION sequencing data and to generate *de novo* assemblies, which were error-corrected using short-read Illumina data and the assembly polisher tool. Alignments of the fully reconstructed plasmid sequences were visualized and annotated using Geneious Prime (v. 2023.0.4).

For plasmid characterization, a hybrid assembly was generated using QIAGEN CLC Genomics Workbench (version 23.0). Comparison analyses were performed using Geneious Prime (v. 2023.0.4) and BLAST Ring Generator (BRIG v. 0.95) to compare the circularized plasmids from this study with similar plasmids deposited in the NCBI database. Prokka (v. 1.14.6) and reference sequences were used for preliminary annotation, and the coding sequences (CDS) were manually curated.

## 3 Results

During the 2-year study period (January 2020 to January 2022), the incidence rates of MNSSm ranged from 0 to 1.39 cases/1,000 patient-days, with a median of 1.14 cases/1,000 patient-days. The highest rates were observed in December 2020, January 2021, February 2021, and March 2021 with 0.24, 0.19, 0.35, and 1.39 cases/1000 patient-days, respectively. The incidence curve (Supplementary Figure S1) revealed that the outbreak began in December 2020 and concluded in November 2021. Clinical data indicated that 77.65% (132/170) of the isolates were recovered from COVID-19-positive patients. Among these patients, the majority (83%; 109/132) were admitted to the intensive care unit (ICU).

The MNSSm isolates were predominantly obtained from tracheal aspirate samples (77%; 131/170). High resistance rates were observed for cefepime (100%), ceftazidime (98.2%), gentamicin (94.4%), ciprofloxacin (93.6%), sulfamethoxazole-trimethoprim (73.8%), and tigecycline (73.8%) (Supplementary Table S2). Susceptibility to amikacin was observed in 51.2% of the isolates. The MICs for meropenem (4.0–250.0 μg/mL), ceftazidime-avibactam (0.5–256 μg/mL), and meropenem-vaborbactam (0.06–8 μg/mL) are presented in Table 1. The MIC₅₀/MIC₉₀ values for meropenem, ceftazidime-avibactam, and meropenem-vaborbactam were 8.0/256, 0.5/8, and 0.125/4 μg/mL, respectively. We successfully recovered 166 out of 170 MNSSm isolates for carbapenemase gene detection. The *bla*_KPC_ gene was the most prevalent carbapenemase gene (92.2%, 153/166), followed by *bla*_NDM-1_ (3.6%; 6/166).

**Table 1:** Clinical characteristics of the isolates for phylogenetic analysis.

Isolate number	Date	Material	Admission unit	Age	COVID-19	Death within 30 days	TYG MIC (μg/mL)	MPM MIC (μg/mL)	CZA MIC (μg/mL)	MRV MIC (μg/mL)	Gene carbapenemase	Clade
8	03/12/2020	Catheter tip	Surgical unit	53	Yes	No	2	8	0.5	1	KPC	A
9	06/12/2020	Tracheal aspirate	ICU 7B	61	Yes	No	0.5	256	8	2	KPC	A
12	29/12/2020	Tracheal aspirate	ICU 7C	66	Yes	Yes	1	16	0.25	0.06	KPC	A
13	02-01-2021	Tracheal aspirate	ICU 7B	61	Yes	Yes	1	64	1	0.25	KPC	A
15	07-01-2021	Tracheal aspirate	ICU A COVID	81	Yes	Yes	1	4	0.5	0.06	KPC	A
17	01-02-2021	Tracheal aspirate	ICU 7C	60	Yes	Yes	1	256	8	1	KPC	A
18	03-02-2021	Blood culture	ICU 7B	30	No	Yes	1	8	0.5	0.125	KPC	A
29	06-03-2021	Tracheal aspirate	ICU 7B	52	Yes	No	2	256	1	4	KPC	A
31	10-03-2021	Tracheal aspirate	ICU 2	66	Yes	Yes	0.5	256	8	4	KPC	A
37	16-03-2021	Tracheal aspirate	ICU 6A	27	Yes	Yes	1	8	0.5	0.06	KPC	A
38	16-03-2021	Tracheal aspirate	ICU 2	60	Yes	No	0.5	256	4	4	KPC	A
41	18-03-2021	Tracheal aspirate	ICU 1	62	Yes	Yes	1	16	0.5	0.125	KPC	A
44	24-03-2021	Tracheal aspirate	ICU 2	62	No	Yes	1	4	0.25	0.06	KPC	A
52	30-03-2021	Tracheal aspirate	ICU 7E	52	Yes	No	1	256	8	2	KPC	A
53	30-03-2021	Tracheal aspirate	ICU 7E	55	Yes	No	1	256	0.5	0.125	KPC	A
66	05-04-2021	Tracheal aspirate	Cardiovascular ICU	69	Yes	Yes	1	8	256	2	KPC	A
67	05-04-2021	Tracheal aspirate	ICU 6A	40	Yes	No	1	8	0.5	0.06	KPC	A
68	06-04-2021	Tracheal aspirate	ICU 2	66	Yes	No	1	16	0.5	0.06	KPC	A
69	06-04-2021	Blood culture	ICU 7D	74	Yes	Yes	2	16	256	4	KPC	A
73	06-04-2021	Tracheal aspirate	ICU 2	71	Yes	Yes	2	8	0.25	NA	KPC	A
75	11-04-2021	Tracheal aspirate	ICU 6B	38	Yes	No	0.5	256	8	4	KPC	A
54	10-05-2021	Tracheal aspirate	ICU 2	59	Yes	No	1	8	0.25	NA	KPC	A
55	11-05-2021	Tracheal aspirate	ICU 7A	61	Yes	Yes	1	8	0.5	0.125	KPC	A
57	21-05-2021	Bronchoalveolar lavage	ICU 7D	52	Yes	No	0.5	256	32	1	KPC	A
103	25-05-2021	Tracheal aspirate	ICU 7A	71	Yes	Yes	1	8	0.5	0.06	KPC	A
102	28-05-2021	Blood culture	ICU 6C	56	Yes	No	0.5	8	0.25	0.06	KPC	A
108	05-06-2021	Bronchoalveolar lavage	ICU 6B	40	Yes	Yes	0.5	4	1	0.06	KPC	A
111	08-06-2021	Tracheal aspirate	ICU 7B	60	Yes	No	1	8	0.5	0.06	KPC	A
114	14-06-2021	Sputum	ICU 6B	61	Yes	Yes	1	16	NA	NA	KPC	A
115	16-06-2021	Tracheal aspirate	ICU 7C	48	Yes	Yes	0.5	8	NA	NA	KPC	A
120	19-06-2021	Tracheal aspirate	ICU 7A	53	Yes	No	2	16	NA	NA	KPC	A
119	21-06-2021	Tracheal aspirate	ICU 6 E	44	Yes	No	1	8	1	0.06	KPC	A
128	05-07-2021	Sputum	Surgical unit	64	Yes	No	4	32	NA	NA	KPC	A
129	10-07-2021	Tracheal aspirate	ICU 7C	73	Yes	Yes	1	8	0.5	0.06	KPC	A
133	15-07-2021	Tracheal aspirate	ICU 6D	50	Yes	Yes	1	4	0.5	0.125	KPC	A
131	16-07-2021	Blood culture	ICU 7C	29	Yes	Yes	1	256	1	4	KPC	A
134	18-07-2021	Tracheal aspirate	ICU 6B	35	Yes	Yes	0.5	8	0.5	0.125	KPC	A
136	18-07-2021	Tracheal aspirate	ICU 6D	52	Yes	Yes	0.5	4	0.5	0.5	KPC	A
139	26-07-2021	Bronchoalveolar lavage	ICU 6D	28	Yes	No	0.5	16	NA	NA	KPC	A
140	27-07-2021	Lung secretion	ICU 7B	43	Yes	No	0.5	32	NA	NA	KPC	A
146	19-08-2021	Tracheal aspirate	ICU 6D	71	Yes	No	0.5	16	NA	NA	KPC	A
149	08-09-2021	Sputum	ICU 2	21	Yes	Yes	8	16	NA	NA	KPC	A
151	26-09-2021	Tracheal aspirate	ICU 6E	71	Yes	Yes	1	4	0.5	0.125	KPC	A
153	08-10-2021	Tracheal aspirate	ICU 6D	35	Yes	No	1	8	NA	NA	KPC	A
154	16-10-2021	Tracheal aspirate	ICU 2	50	No	No	0.5	256	1	1	KPC	A
159	06-11-2021	Tracheal aspirate	ICU 6C	40	Yes	Yes	1	8	NA	NA	KPC	A
160	06-11-2021	Tracheal aspirate	ICU 1	68	No	Yes	1	8	0.5	0.06	KPC	A
163	08-11-2021	Tracheal aspirate	ICU 1	68	No	Yes	1	16	NA	NA	KPC	A
169	30-11-2021	Bronchoalveolar lavage	ICU 3	66	No	Yes	0.5	16	NA	NA	KPC	A
167	17-11-2021	Sputum	ICU 6C	65	Yes	Yes	1	4	256	1.5	NDM	B
s17	28//09/2021	Bronchoalveolar lavage	ICU 1	42	No	No	NA	NA	NA	NA	None	B

ICU, Intensive Care Unit; S, susceptible; I,intermediary; R, resistant; NA, not available; AK, amikacin; SMZ+TMP, sulfamethoxazole-thyrotropin; FEP, cefepime; CIP, ciprofloxacin; NOR, norfloxacin; CN, gentamicin; TZP, piperacillin-tazobactam; MPM, meropenem; CZA, ceftazidime-avibactam; MRV, meropenem-vaborbactam; NA, not realized. MIC50/MIC90 of MPM, CZA and MRV were 8.0/256, 0.5/8, and 0.125/4 μg/mL, respectively.

**Figure 1:**
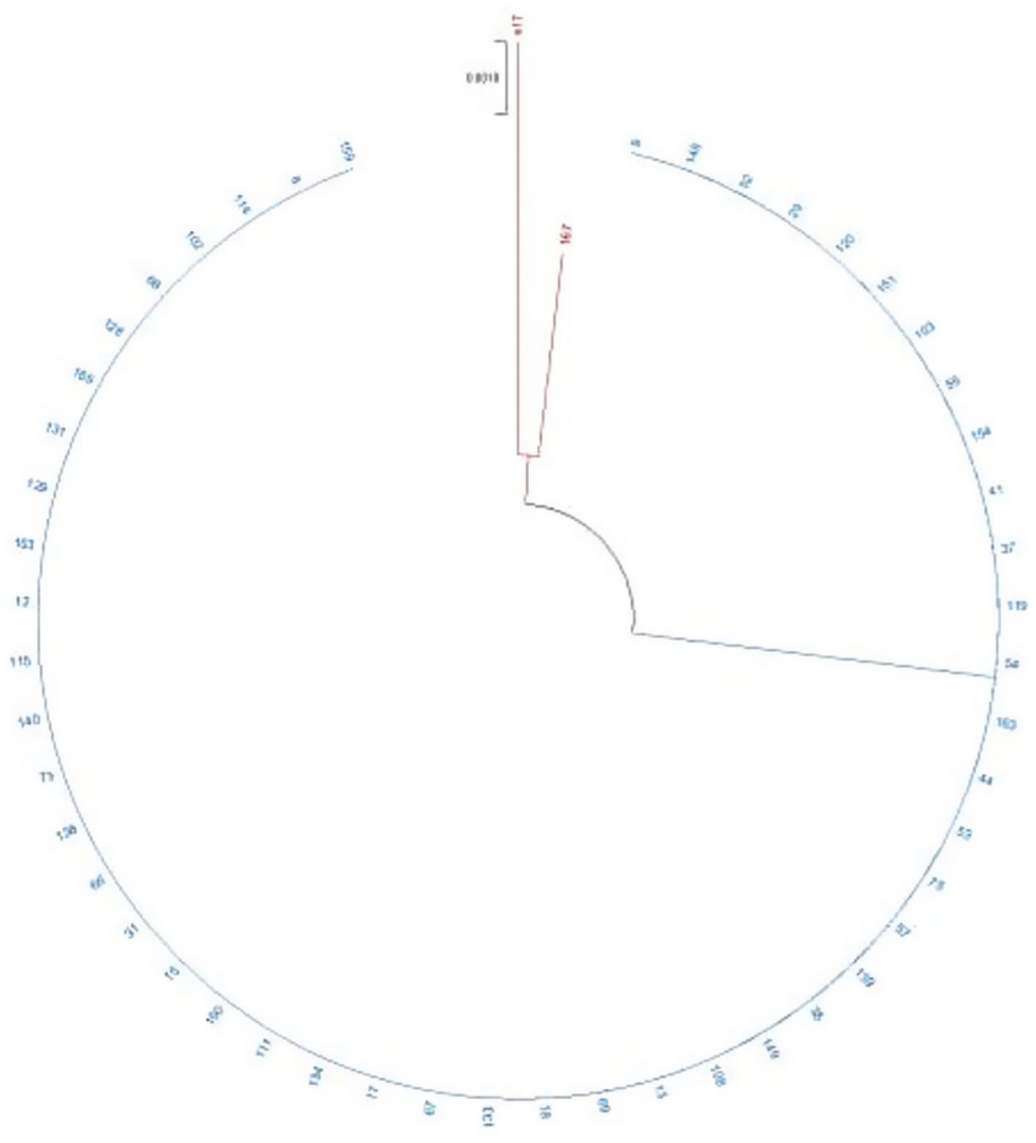
Phylogenetic analysis of the *Serratia marcescens* isolates. The phylogenetic tree was inferred using the maximum likelihood (ML) method. The bootstrap test was based on 1,000 replicates. Branches in red represent clade A, and branches in blue represent clade B. Branch lengths are shown above the branches in black. S17 is the meropenem-susceptible isolate obtained during the outbreak period.

For the SLST method using the flagellin gene, eight polymorphic sites were identified in the reference sequences, and a 353 bp DNA sequence was sufficient to resolve all previously reported phylogenetic clades (Table 1). This sequence was designated as the *fliC* gene typing region. Of the 69 isolates amplified by PCR for Sanger sequencing, high-quality sequence data were obtained for 50 isolates (Supplementary Figure S2). Phylogenetic analysis grouped these isolates into two distinct clades: Clade B comprised all *bla*_KPC-2-_positive isolates (49/50), while Clade A contained the single meropenem-susceptible isolate, which was closely related to a *bla*_NDM-1-_positive isolate (Figure 1). WGS revealed that the *Serratia marcescens* isolate GSMA0007 belongs to sequence type 807 (ST807).

**Figure 2:**
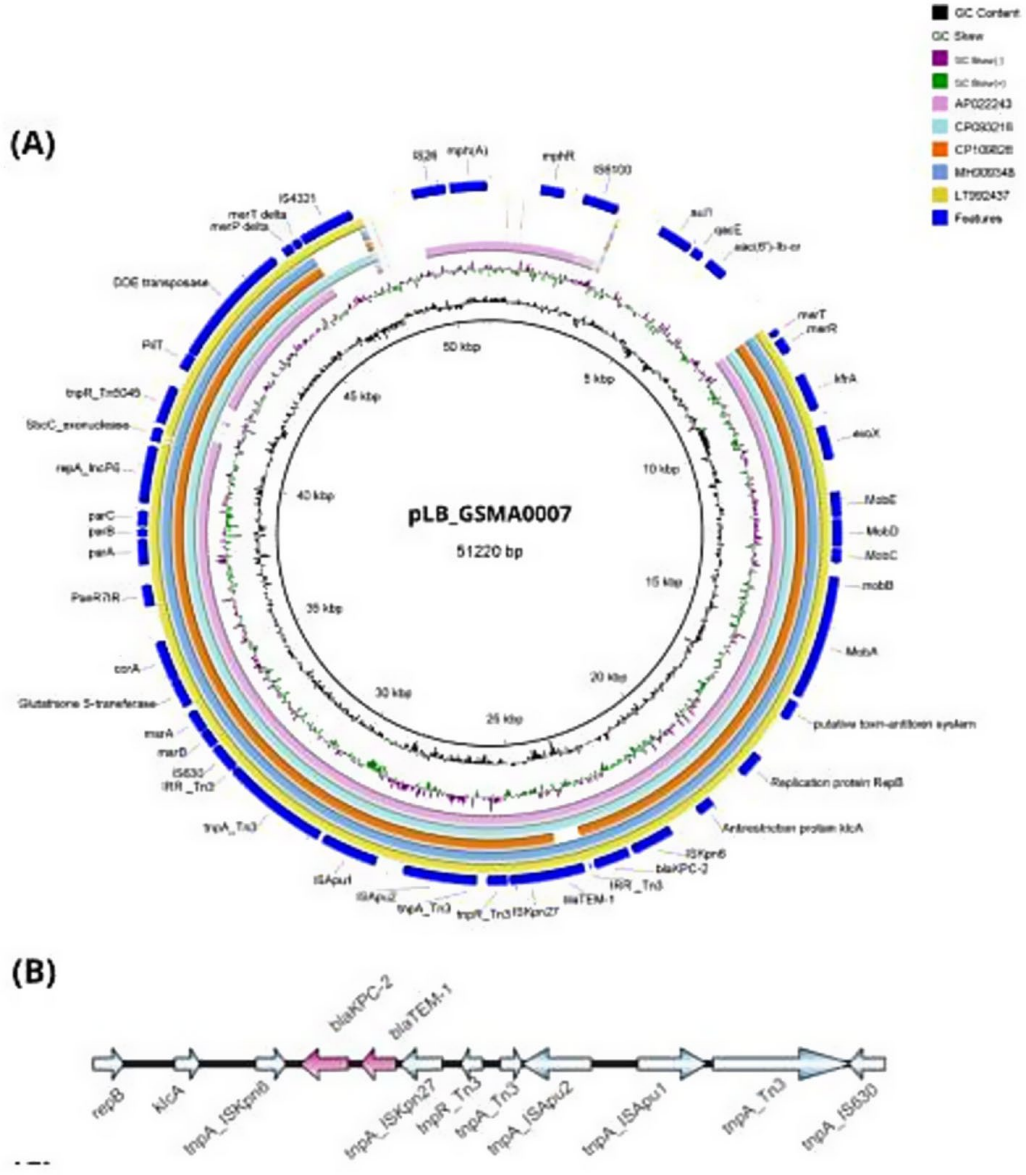
Comparisons among the plasmids belonging to the IncP6 group harboring *bla*_KPC-2_. **(A)** Circular alignment of pLB_GSMA0007 (51,220 kb) with other sequences previously deposited in Genbank (AP022243, CP093216, CP109826, MH909348, LT992437), generated using BLAST Ring Image Generator (BRIG). The inner circles represent the assembly of pLB_GSMA0007, GC content (black), and GC skew (dark green and purple). **(B)** Genetic environment of *bla*_KPC-2_ in pLB_GSMA0007, generated using IBS 2.0. Resistance genes are represented in pink (for details regarding the isolates, see Supplementary Table S3).

The *bla*_KPC-2_ gene was located on a plasmid with 99.93% identity and 83% coverage to pWP8-S18-CRE-01_2 (GenBank accession number AP022243.1). PlasmidFinder identified the incompatibility group as IncP6, with 99.8% identity and 100% coverage. The complete circularized IncP6 plasmid exhibited a GC content of 58.3% and measured 51,220 kb in size; it was designated pLB_GSMA0007 (accession number CP130614 and CP130615). A graphical comparison of the IncP6 plasmids harboring *bla*KPC-2 is presented in Figure 2. The *bla*_KPC-2_ gene was inserted within a classical Tn3-family transposon alongside other antibiotic resistance genes, including *bla*_TEM-1_, mph(A), qacE, sul1, and aac(6′)-lb-cr, which confer resistance to cephalosporins, macrolides, chlorhexidine and benzalkonium chloride, sulfamethoxazole, fluoroquinolones, and aminoglycosides, respectively. Complete information regarding the whole genome analysis is provided in Supplementary Table S3.

## 4 Discussion

The COVID-19 pandemic significantly disrupted hospital settings worldwide, increasing the demand for ICU beds, medical supplies, and healthcare workers. This surge severely impacted hospital healthcare systems. The prolonged and complex course of SARS-CoV-2 infections weakened surveillance measures for multi-drug resistant (MDR) organisms, creating favorable conditions for hospital-acquired infections (HAIs) (Falcone et al., 2022; Kozłowski et al., 2022). Extensive antimicrobial exposure, prolonged hospitalization, use of invasive devices, and compromised host immunity are considered the primary factors associated with antimicrobial resistance development (De Waele et al., 2020). According to data from our institution, the highest number of hospitalized COVID-19 patients was recorded between 19 February 2021 and 17 March 2021 (Martins et al., 2021) Notably, the highest incidence density of MNSSm was observed in March 2021. During the outbreak in our institution, meropenem was the fourth most commonly used antimicrobial among COVID-19 patients (Silva et al., 2020). Its consumption, measured in days of therapy (DOT) per 1,000 patient-days, was higher in 2021 than in 2020 (101.4 vs. 90.9, respectively) (data not shown).

*S. marcescens* has long been recognized as a cause of nosocomial outbreaks. During the pandemic, several hospital outbreaks of carbapenem-resistant *S. marcescens* were linked to COVID-19 dedicated units (Vera-Leiva et al., 2017; World Health Organization, 2017; Nedeljković et al., 2021). These outbreaks were due to *S. marcescens* carrying the *bla*_KPC-2_ gene, primarily located on plasmid groups IncA/C and IncN (Prado et al., 2022). The transmission of *S. marcescens* in healthcare settings is often associated with direct patient contact, contaminated medical equipment, and healthcare personnel. In our outbreak, the predominance of cases among ICU inpatients suggests a likely role of invasive procedures, such as mechanical ventilation and central venous catheters, as potential facilitators of bacterial spread. In addition, environmental reservoirs, including sinks and disinfectant solutions, have been previously implicated in *S. marcescens* outbreaks. Upon identifying the outbreak, immediate infection control measures were implemented, including cohorting of infected patients, enhanced hand hygiene reinforcement among healthcare workers, and decontamination of high-touch surfaces.

Treatment of infections caused by carbapenem-resistant *S. marcescens* is challenging due to this bacterium’s intrinsic resistance to polymyxins. Newer beta-lactam/beta-lactamase inhibitor combinations may be effective against carbapenem-resistant *S. marcescens* but only when resistance is mediated by serine carbapenemases rather than metallo-carbapenemases. Therefore, identification of bacterial resistance mechanisms plays a crucial role in determining appropriate clinical treatment for patients with carbapenem-resistant infections. Our findings demonstrated that the *S. marcescens* isolates carrying *bla*_KPC_ were susceptible to ceftazidime-avibactam and meropenem-vaborbactam, consistent with previous reports (Prado et al., 2022).

Evaluating clonal relatedness of isolates during an outbreak is essential, with PFGE and cgMLST schemes being the most common typing methods. However, developing faster, reliable, and cost-effective methods remains necessary. Recently, various typing approaches using single- or double-locus sequence typing have been proposed (Weissman et al., 2012; Pournaras et al., 2014; Fernández-Huerta et al., 2020; Magalhães et al., 2020) to enable rapid evaluation of outbreak isolates. In this study, we evaluated a rapid approach to characterize an *S. marcescens* outbreak using a 353 bp region of the *fliC* gene. This gene encodes flagellin, the primary protein constituting the flagellar structure in various bacterial species. The flagellin sequence contains highly conserved regions across species, as well as a hypervariable central region (Nedeljković et al., 2021), making the *fliC* gene an interesting molecular marker for typing. Using this gene, our phylogenetic analysis clustered all *bla*KPC-2-positive isolates into the same clade while distinguishing both *bla*NDM-1-positive and meropenem-susceptible isolates. Although this molecular marker produced promising results, it is important to emphasize that confirmation of isolate clonality should utilize more robust methods.

In this study, the *bla*_KPC-2_ gene was carried on an IncP6 incompatibility plasmid of 51,220 kb (pLB_GSMA0007). The genetic environment of the carbapenemase gene harbored a Tn3 transposon formed by IS*Kpn6*/*bla*KPC-2/Δ*bla*TEM-1/IS*Kpn27,* identical to the structure previously reported by Yao et al. (2017). While the genetic context of *bla*_KPC-2_ varies across different plasmids, the most common transposon in Brazil is Tn4401, which has been responsible for the widespread dissemination of this gene in the country (Vera-Leiva et al., 2017). IncP6 plasmids carrying *bla*_KPC-2_ have rarely been reported, and to the best of our knowledge, this is the first report of an IncP6 plasmid from a clinical isolate in Brazil.

Our findings demonstrate that an outbreak of clonal-related carbapenem-resistant *S. marcescens* occurred during the COVID-19 pandemic, primarily affecting ICU inpatients. The spread of the resistance gene was facilitated by an IncP6 plasmid containing *bla*_KPC-2_, reported here for the first time from a clinical isolate in Brazil. In addition, our approach using the *fliC* gene for SLST successfully enabled molecular characterization of the *S. marcescens* outbreak. This method is particularly valuable given that whole-genome sequencing, while considered the gold standard, is not always feasible. The SLST method represents a promising tool for genomic surveillance due to its lower cost and faster turnaround time.

## Data Availability

The datasets presented in this study can be found in online repositories. The names of the repository/repositories and accession number(s) can be found in the article/supplementary material.

## Footnotes

^1^ https://pubmlst.org/bigsdb?db=pubmlst_serratia_seqdef

## Supplementary material

SUPPLEMENTARY FIGURE 1 Incidence rate of the 170 MNSSm isolates from January 2020 to January 2022. Incidence rate was calculated as MNSSm isolates per 1000 Patient Days.

SUPPLEMENTARY FIGURE 2 Alignment of *fliC* gene (350 bp fragment). This MAFFT alignment shows the identity of the fliC gene fragment among outbreak isolates and an outgroup isolate, used to build the phylogenetic tree (Figure 1). The colours yellow (G), green (T), red (A) and blue (C) represent the SNPs in each position.

## References

[ref1] Barberino M. G. Cruvinel S. d. A. Faria C. Salvino M. A. Silva M. d. O. (2018). Isolation of blaNDM-producing Enterobacteriaceae in a public hospital in Salvador, Bahia, Brazil. Braz. J. Infect. Dis. 22, 47–50. doi: 10.1016/j.bjid.2017.10.002, PMID: 29144958 PMC9425538

[ref2] Cayô R. Leme R. C. P. Streling A. P. Matos A. P. Nodari C. S. Chaves J. R. E.. (2017). *Serratia marcescens* harboring SME-4 in Brazil: a silent threat. Diagn. Microbiol. Infect. Dis. 87, 357–358. doi: 10.1016/j.diagmicrobio.2017.01.008, PMID: 28159445

[ref4] da Silva K. E. Rossato L. Jorge S. de Oliveira N. R. Kremer F. S. Campos V. F.. (2021). Three challenging cases of infections by multidrug-resistant *Serratia marcescens* in patients admitted to intensive care units. Braz. J. Microbiol. 52, 1341–1345. doi: 10.1007/s42770-021-00477-4, PMID: 33829377 PMC8324748

[ref5] Dashti A. A. Jadaon M. M. Abdulsamad A. M. Dashti H. M. (2009). Heat treatment of bacteria: a simple method of DNA extraction for molecular techniques. Kuw. Med. J. 41, 117–122.

[ref6] De Waele J. J. Boelens J. Leroux-Roels I. (2020). Multidrug-resistant bacteria in ICU: fact or myth. Curr. Opin. Anaesthesiol. 33, 156–161. doi: 10.1097/ACO.0000000000000830, PMID: 31904697

[ref7] EUCAST Breakpoint tables for interpretation of MICs and zone diameters, version 14.0. EUCAST (2024). Available online at: https://www.eucast.org/clinical_breakpoints/

[ref8] Eucast: MIC Determination (2025). Available at: https://www.eucast.org/ast_of_bacteria/mic_determination (Accessed October 24, 2024).

[ref9] Falcone M. Suardi L. R. Tiseo G. Galfo V. Occhineri S. Verdenelli S.. (2022). Superinfections caused by carbapenem-resistant Enterobacterales in hospitalized patients with COVID-19: a multicentre observational study from Italy (CREVID study). JAC Antimicrob. Resist. 4:dlac064. doi: 10.1093/jacamr/dlac064, PMID: 35719203 PMC9201238

[ref10] Fernández-Huerta M. Serra-Pladevall J. Esperalba J. Moreno-Mingorance A. Fernández-Naval C. Barberá M.-J.. (2020). Single-locus-sequence-based typing of the mgpB gene reveals transmission dynamics in *Mycoplasma genitalium*. J. Clin. Microbiol. 58:e01886. doi: 10.1128/JCM.01886-19, PMID: 31941694 PMC7098777

[ref11] Hamers R. L. Cassini A. Asadinia K. S. Bertagnolio S. (2022). Developing a priority global research agenda for antimicrobial resistance in the human health sector: protocol for a scoping review. BMJ Open 12:e060553. doi: 10.1136/bmjopen-2021-060553, PMID: 35654465 PMC9163534

[ref12] Iguchi A. Nagaya Y. Pradel E. Ooka T. Ogura Y. Katsura K.. (2014). Genome evolution and plasticity of *Serratia marcescens*, an important multidrug-resistant nosocomial pathogen. Genome Biol. Evol. 6, 2096–2110. doi: 10.1093/gbe/evu160, PMID: 25070509 PMC4231636

[ref13] Jimenez A. Abbo L. M. Martinez O. Shukla B. Sposato K. Iovleva A.. (2020). KPC-3–producing *Serratia marcescens* outbreak between acute and long-term care facilities, Florida, USA. Emerg. Infect. Dis. 26, 2746–2750. doi: 10.3201/eid2611.202203, PMID: 33079055 PMC7588513

[ref14] Kalyaanamoorthy S. Minh B. Q. Wong T. K. F. von Haeseler A. Jermiin L. S. (2017). ModelFinder: fast model selection for accurate phylogenetic estimates. Nat. Methods 14, 587–589. doi: 10.1038/nmeth.4285, PMID: 28481363 PMC5453245

[ref15] Katoh K. Standley D. M. (2013). MAFFT multiple sequence alignment software version 7: improvements in performance and usability. Mol. Biol. Evol. 30, 772–780. doi: 10.1093/molbev/mst010, PMID: 23329690 PMC3603318

[ref16] Kearse M. Moir R. Wilson A. Stones-Havas S. Cheung M. Sturrock S.. (2012). Geneious basic: an integrated and extendable desktop software platform for the organization and analysis of sequence data. Bioinformatics 28, 1647–1649. doi: 10.1093/bioinformatics/bts199 22543367 PMC3371832

[ref17] Kozłowski B. Kubiak-Pulkowska J. Pałka J. Bożiłow D. Zając M. Deptuła A. (2022). Healthcare-associated infections in COVID-19 ICU patients - two-Centre study. Cent. Eur. J. Public Health 30, 196–200. doi: 10.21101/cejph.a7135, PMID: 36239369

[ref18] Kumar S. Stecher G. Li M. Knyaz C. Tamura K. (2018). MEGA X: molecular evolutionary genetics analysis across computing platforms. Mol. Biol. Evol. 35, 1547–1549. doi: 10.1093/molbev/msy096, PMID: 29722887 PMC5967553

[ref9001] Martins A. Zavascki A. Wink P. Volpato F. Monteiro F. Rosset C.. (2021). Detection of SARS-CoV-2 lineage P.1 in patients from a region with exponentially increasing hospitalisation rate, February 2021, Rio Grande do Sul, Southern Brazil. Euro Surveill. 26. doi: 10.2807/1560-7917.ES.2021.26.12.2100276 PMC7995561 33769251

[ref19] Magalhães B. Valot B. Abdelbary M. M. H. Prod’hom G. Greub G. Senn L.. (2020). Combining standard molecular typing and whole genome sequencing to investigate *Pseudomonas aeruginosa* epidemiology in intensive care units. Front. Public Health 8:3. doi: 10.3389/fpubh.2020.00003 32047733 PMC6997133

[ref20] Martineau C. Li X. Lalancette C. Perreault T. Fournier E. Tremblay J.. (2018). *Serratia marcescens* outbreak in a neonatal intensive care unit: new insights from next-generation sequencing applications. J. Clin. Microbiol. 56:e00235. doi: 10.1128/JCM.00235-18, PMID: 29899005 PMC6113457

[ref21] Monteiro J. Widen R. H. Pignatari A. C. C. Kubasek C. Silbert S. (2012). Rapid detection of carbapenemase genes by multiplex real-time PCR. J. Antimicrob. Chemother. 67, 906–909. doi: 10.1093/jac/dkr563, PMID: 22232516

[ref9002] Moradigaravand D. Boinett C. J. Martin V. Peacock S. J. Parkhill J. (2016). Recent independent emergence of multiple multidrug-resistant Serratia marcescens clones within the United Kingdom and Ireland. Genome Res. 26, 1101–9. doi: 10.1101/gr.205245.116 27432456 PMC4971767

[ref22] Muyldermans A. Crombé F. Bosmans P. Cools F. Piérard D. Wybo I. (2021). *Serratia marcescens* outbreak in a neonatal intensive care unit and the potential of whole-genome sequencing. J. Hosp. Infect. 111, 148–154. doi: 10.1016/j.jhin.2021.02.006, PMID: 33581246

[ref23] Nedeljković M. Sastre D. E. Sundberg E. J. (2021). Bacterial flagellar filament: a supramolecular multifunctional nanostructure. Int. J. Mol. Sci. 22:7521. doi: 10.3390/ijms22147521, PMID: 34299141 PMC8306008

[ref24] Nguyen L.-T. Schmidt H. A. Von Haeseler A. Minh B. Q. (2015). IQ-TREE: a fast and effective stochastic algorithm for estimating maximum-likelihood phylogenies. Mol. Biol. Evol. 32, 268–274. doi: 10.1093/molbev/msu300, PMID: 25371430 PMC4271533

[ref9003] Nodari C. S. Siebert M. Matte U. D. S. Luís Barth A. (2017). Draft genome sequence of a GES-5-producing Serratia marcescens isolated in southern Brazil. Braz J Microbiol. 48, 191–192. doi: 10.1016/j.bjm.2016.08.002 27932081 PMC5470340

[ref25] NOTA TÉCNICA No. 74/2022-CGLAB/DAEVS/SVS/MS-Agência Nacional de Vigilância Sanitária-Anvisa (2025). Available at: https://www.gov.br/anvisa/pt-br/centraisdeconteudo/publicacoes/servicosdesaude/notas-tecnicas/notas-tecnicas-vigentes/nota-tecnica-no-74-2022-cglab-daevs-svs-ms/view (Accessed October 24, 2024).

[ref26] Pintado V. Ruiz-Garbajosa P. Escudero-Sanchez R. Gioia F. Herrera S. Vizcarra P.. (2022). Carbapenemase-producing Enterobacterales infections in COVID-19 patients. Infect. Dis. 54, 36–45. doi: 10.1080/23744235.2021.1963471, PMID: 34382910 PMC8425444

[ref27] Pournaras S. Gogou V. Giannouli M. Dimitroulia E. Dafopoulou K. Tsakris A.. (2014). Single-locus-sequence-based typing of blaOXA-51-like genes for rapid assignment of *Acinetobacter baumannii* clinical isolates to international clonal lineages. J. Clin. Microbiol. 52, 1653–1657. doi: 10.1128/JCM.03565-13, PMID: 24622099 PMC3993655

[ref28] Prado G. Mendes E. T. Martins R. C. R. Perdigão-Neto L. V. Freire M. P. Marchi A. P.. (2022). Phenotypic and genotypic characteristics of a carbapenem-resistant *Serratia marcescens* cohort and outbreak: describing an opportunistic pathogen. Int. J. Antimicrob. Agents 59:106463. doi: 10.1016/j.ijantimicag.2021.106463, PMID: 34715332

[ref29] Šiširak M. (2013). An outbreak of multidrug-resistant *Serratia marcescens*: the importance of continuous monitoring of nosocomial infections. Acta Med. Acad. 42, 25–31. doi: 10.5644/ama2006-124.67, PMID: 23735063

[ref9004] Silva C. F. da Deutschendorf C. Nagel F. M. Dalmora C. H. Santos R. P. dos Lisboa T. C. (2020). Impact of the pandemic on antimicrobial consumption patterns. Infect. Control Hosp. Epidemiol. 1, 1170–1172. doi: 10.1017/ice.2020.1227 PMC7542322 32962779

[ref30] Streling A. P. Barbosa P. P. Marcondes M. F. Nicoletti A. G. Picão R. C. Pinto E. C.. (2018). Genetic and biochemical characterization of GES-16, a new GES-type β-lactamase with carbapenemase activity in *Serratia marcescens*. Diagn. Microbiol. Infect. Dis. 92, 147–151. doi: 10.1016/j.diagmicrobio.2018.05.003, PMID: 29861147

[ref31] Vera-Leiva A. Barría-Loaiza C. Carrasco-Anabalón S. Lima C. Aguayo-Reyes A. Domínguez M.. (2017). KPC: *Klebsiella pneumoniae* carbapenemasa, principal carbapenemasa en enterobacterias. Rev. Chil. Infectol. 34, 476–484. doi: 10.4067/S0716-10182017000500476, PMID: 29488590

[ref32] Weissman S. J. Johnson J. R. Tchesnokova V. Billig M. Dykhuizen D. Riddell K.. (2012). High-resolution two-locus clonal typing of Extraintestinal pathogenic *Escherichia coli*. Appl. Environ. Microbiol. 78, 1353–1360. doi: 10.1128/AEM.06663-11, PMID: 22226951 PMC3294456

[ref33] World Health Organization. (2017). Guidelines for the prevention and control of carbapenem-resistant *Enterobacteriaceae*, *Acinetobacter baumannii* and *Pseudomonas aeruginosa* in health care facilities. Geneva: World Health Organization. Available online at: https://apps.who.int/iris/handle/10665/259462 (Accessed September 27, 2022). 29630191

[ref34] Yao Y. Lazaro-Perona F. Falgenhauer L. Valverde A. Imirzalioglu C. Dominguez L.. (2017). Insights into a novel blaKPC-2 -encoding IncP-6 plasmid reveal carbapenem-resistance circulation in several Enterobacteriaceae species from wastewater and a hospital source in Spain. Front. Microbiol. 8:1143. doi: 10.3389/fmicb.2017.01143, PMID: 28702005 PMC5487458

[ref35] Zhang R. Zhou H. W. Cai J. C. Chen G.-X. (2007). Plasmid-mediated carbapenem-hydrolysing beta-lactamase KPC-2 in carbapenem-resistant *Serratia marcescens* isolates from Hangzhou, China. J. Antimicrob. Chemother. 59, 574–576. doi: 10.1093/jac/dkl541, PMID: 17251347

[ref36] Zingg W. Soulake I. Baud D. Huttner B. Pfister R. Renzi G.. (2017). Management and investigation of a *Serratia marcescens* outbreak in a neonatal unit in Switzerland – the role of hand hygiene and whole genome sequencing – R1, ARIC-D-17-00143. Antimicrob. Resist. Infect. Control 6:125. doi: 10.1186s13756-017-0285-x 29238572 10.1186/s13756-017-0285-x PMC5725813

