# Supplementary figures and images for: Molecular epidemiology of a carbapenem-resistant Serratia marcescens outbreak during the COVID-19 pandemic
Source: Front Microbiol. 2025 Jul 2;16:1525543. doi: 10.3389/fmicb.2025.1525543 (PMC12263918; doi:10.3389/fmicb.2025.1525543)

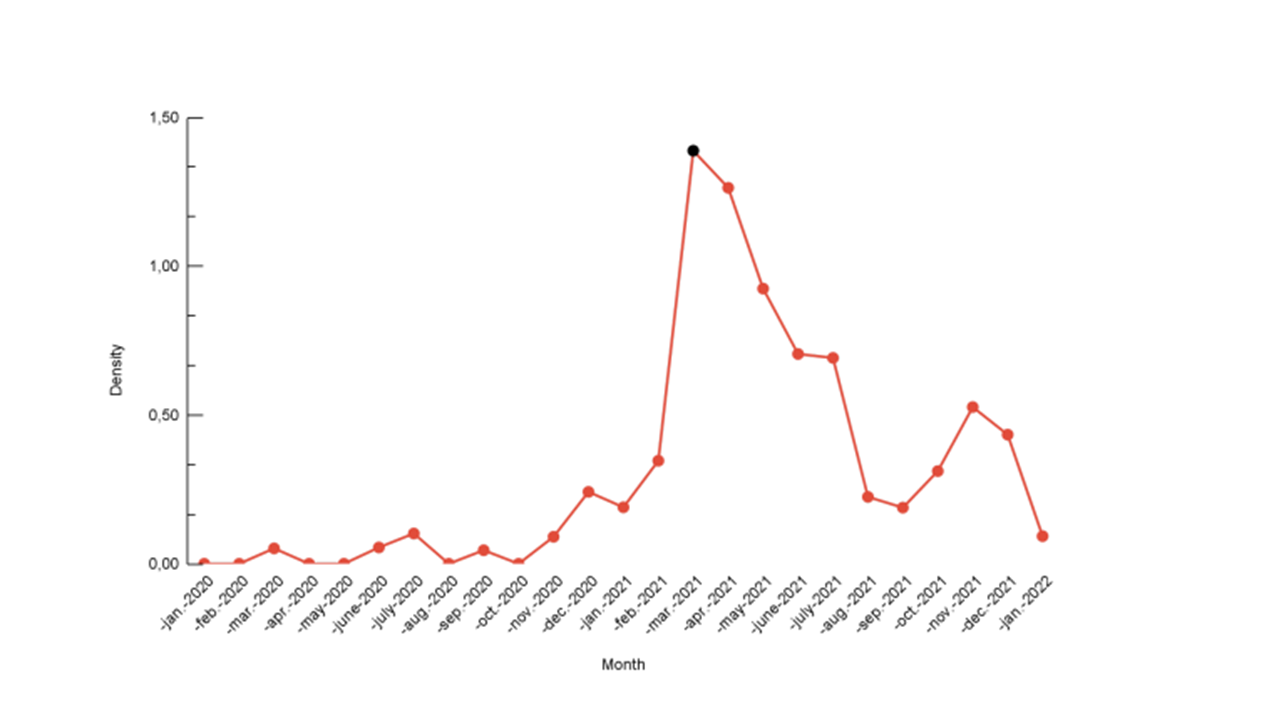

Supplement: SUPPLEMENTARY FIGURE 1 — Incidence rate of the 170 MNSSm isolates from January 2020 to January 2022. Incidence rate was calculated as MNSSm isolates per 1000 Patient Days. [file Image_1.tif]

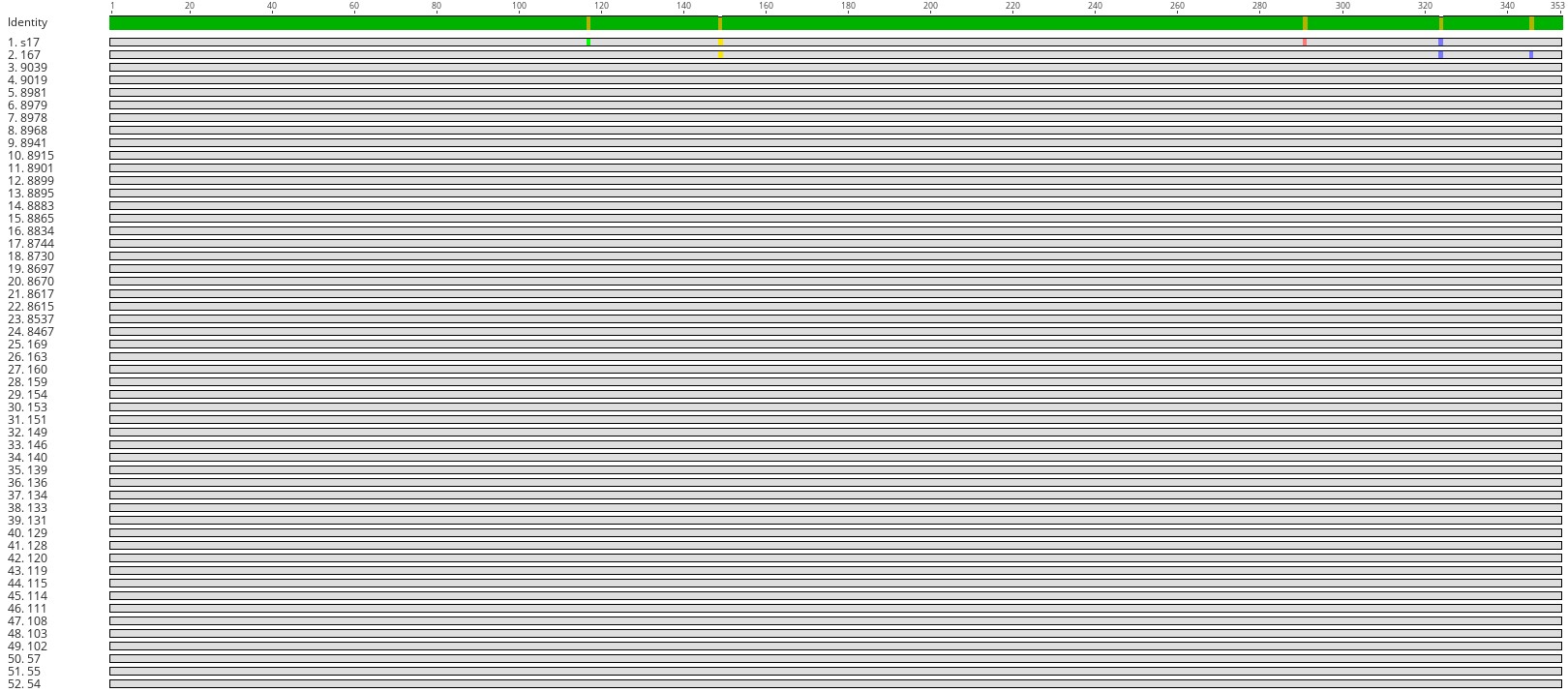

Supplement: SUPPLEMENTARY FIGURE 2 — Alignment of fliC gene (350 bp fragment). This MAFFT alignment shows the identity of the fliC gene fragment among outbreak isolates and an outgroup isolate, used to build the phylogenetic tree (Figure 1). The colours yellow (G), green (T), red (A) and blue (C) represent the SNPs in each position. [file Image_2.jpg]
