# Supplementary material for: Molecular epidemiology of a carbapenem-resistant Serratia marcescens outbreak during the COVID-19 pandemic
Source: Front Microbiol. 2025 Jul 2;16:1525543. doi: 10.3389/fmicb.2025.1525543 (PMC12263918; doi:10.3389/fmicb.2025.1525543)
Supplement: Supplementary file 3 [file Table_1.docx]

| **Table S1.** Reference sequences of the flagellin (*fliC*) gene from *S. marcescens* | | | | | | | | |
| --- | --- | --- | --- | --- | --- | --- | --- | --- |
| **Sequence** | **Accession No.ª** | **Strain name^b^** | **Clone^b^** | **Genome size** | **Submitter** | **Submission date** | **ST** | **Reference** |
| SER525_CLONEA | GCF_009938955.1 | SER 525 | CLONE A | 5.3 Mb | University of Pittsburgh | jan. 28, 2020 | 789 | Jimenez A, *et al*. 2020¹ |
| SER505_CLONEA | GCF_010078335.1 | SER 505 | CLONE A | 5.3 Mb | University of Pittsburgh | jan. 31, 2020 | 789 |  |
| SER523_CLONEA | GCF_010092645.1 | SER 523 | CLONE A | 5.3 Mb | University of Pittsburgh | feb. 2, 2020 | 789 |  |
| SER522_CLONEA | GCF_010092675.1 | SER 522 | CLONE A | 5.3 Mb | University of Pittsburgh | feb. 2, 2020 | 789 |  |
| SER518_CLONEA | GCF_010092745.1 | SER 518 | CLONE A | 5.3 Mb | University of Pittsburgh | feb. 2, 2020 | 789 |  |
| SER517_CLONEA | GCF_010092765.1 | SER 517 | CLONE A | 5.3 Mb | University of Pittsburgh | feb. 2, 2020 | 789 |  |
| SER501_CLONEB | GCF_009939185.1 | SER 501 | CLONE B | 5.1 Mb | University of Pittsburgh | jan. 28, 2020 | 789 |  |
| SER514_CLONEB | GCF_010078255.1 | SER 514 | CLONE B | 4.9 Mb | University of Pittsburgh | jan. 31, 2020 | 789 |  |
| SER502_CLONEC | GCF_009939145.1 | SER 502 | CLONE C | 5.2 Mb | University of Pittsburgh | jan. 28, 2020 | 490 |  |
| SER508_CLONEC | GCF_010078355.1 | SER 508 | CLONE C | 5.2 Mb | University of Pittsburgh | jan. 31, 2020 | 490 |  |
| SER500_CLONEC | GCF_010092755.1 | SER 500 | CLONE C | 5.2 Mb | University of Pittsburgh | feb. 2, 2020 | 490 |  |
| SER520_CLONED | GCF_010092665.1 | SER520 | CLONE D | 5.1 Mb | University of Pittsburgh | feb. 2, 2020 | 795 |  |
| H12_UK_2010_1 | GCF_001538465.1 | 2880STDY5683010 | CLONE E | 5.2 Mb | NA | jan. 22, 2016 | 365 | Moradigaravand D, *et al*. 2016² |
| H12_UK_2010_2 | GCF_001539125.1 | 2880STDY5683011 | CLONE E | 5.2 Mb | NA | jan. 22, 2016 | 365 |  |
| H12_UK_2010_3 | GCF_001537445.1 | 2880STDY5683014 | CLONE E | 5.2 Mb | NA | jan. 22, 2016 | 365 |  |
| SM_39 | GCF_000828775.1 | SM39 | Not outbreak related - 1999 clinical isolate, Japan | 5.3 Mb | Frontier Science Research Center, University of Miyazaki | jan. 9, 2014 | 322 | Iguchi A, *et al.* 2014³ |
| Db11 | GCF_000513215.1 | Db11 | Not outbreak related - isolated from Drosophila in 1980, Sweden. | 5.1 Mb | WTSI | nov. 26, 2013 | 306 |  |
| UFRGS_3691F | GCF_001566695 | 3691F | UFRGS | 5.4 Mb | Universidade Federal do Rio Grande do Sul | feb. 23, 2016 | 594/865 | Nodari CS, *et al.* 2017⁴ |
| USP_2013_1 | GCF_003204525.1 | 1756 | HC_SP | 5.4 Mb | University of Sao Paulo | jun. 10, 2018 | 625 | NA |
| USP_2013_2 | GCF_003204645.1 | 1763 | HC_SP | 5.6 Mb | University of Sao Paulo | jun. 10, 2018 | 630 | NA |
| ªAccession Number of sequence  ^b^ The phylogenetic relationship among Clones A, B, C, D, E were evaluated by SNP core genome using RAxML | | | | | | | | |
| Abbreviations: UFRGS, Universidade Federal do Rio Grande do Sul; HC_SP, Hospital das Clínicas de São Paulo; WTSI, Wellcome Trust Sanger Institute; NA, not available. | | | | | | | | |
|  |  |  |  |  |  |  |  |  |
|  |  |  |  |  |  |  |  |  |
| ¹Jimenez A, Abbo LM, Martinez O, et al. KPC-3–Producing *Serratia marcescens* Outbreak between Acute and Long-Term Care Facilities, Florida, USA. Emerging Infectious Diseases. 2020;26(11):2746-2750. doi:10.3201/eid2611.202203. | | | | | | | | |
| ²Moradigaravand D, Boinett CJ, Martin V, *et al*. Recent independent emergence of multiple multidrug-resistant *Serratia marcescens* clones within the United Kingdom and Ireland. Genome Res. 2016 Aug;26(8):1101-9. doi: 10.1101/gr.205245.116. | | | | | | |  |  |
| ³Iguchi A, Nagaya Y, Pradel E, *et al*. Genome evolution and plasticity of *Serratia marcescens*, an important multidrug-resistant nosocomial pathogen. Genome Biol Evol. 2014 Aug;6(8):2096-110. doi: 10.1093/gbe/evu160. PMID: 25070509; PMCID: PMC4231636. | | | | | | | | |
| ⁴Nodari CS, Siebert M, Matte UDS *et al*. Draft genome sequence of a GES-5-producing *Serratia marcescens* isolated in southern Brazil. Braz J Microbiol. 2017 Apr-Jun;48(2):191-192. doi: 10.1016/j.bjm.2016.08.002. Epub 2016 Nov 23. PMID: 27932081; PMCID: PMC5470340. | | | | | | | | |
|  |  |  |  |  |  |  |  |  |
