# Supplementary material for: Molecular epidemiology of a carbapenem-resistant Serratia marcescens outbreak during the COVID-19 pandemic
Source: Front Microbiol. 2025 Jul 2;16:1525543. doi: 10.3389/fmicb.2025.1525543 (PMC12263918; doi:10.3389/fmicb.2025.1525543)
Supplement: Supplementary file 5 [file Table_3.docx]

**Table S3.** Data from whole genome sequence (WGS) provided by Illumina MiSeq

| **ID** | **BioProject ID** | **Collection Date** | **Genome size (bp)** | **Total no of reads** | **Contigs (>200bp)** | **Plasmid (bp)** | **N50 (bp)** | **GC content (%)** | **Antimicrobial Resistance Genes** | | **Contig Position** | **Resistance Phenotype** |
| --- | --- | --- | --- | --- | --- | --- | --- | --- | --- | --- | --- | --- |
| GSMA0007 | PRJNA975155 | 11/06/2021 | 5382116 | 2237138 | 219 | 51220 | 134818 | 59,9 | **Macrolide resistance** | SAT-2 | 119733..120257 | streptothricin |
|  |  |  |  |  |  |  |  |  |  | mphA | 2262..3183 | azithromycin, erythromycin, telithromycin, spiramycin |
|  |  |  |  |  |  |  |  |  | **Antibiotic Inactivation Enzyme** | TEM-1 | 29315..29984 | 1st and 2nd generation cephalosporins and their inhibitors |
|  |  |  |  |  |  |  |  |  |  | KPC-2 | 30106..30987 | 1st, 2nd and 3rd generation cephalosporins and their inhibitors, monobactams and carbapenems |
|  |  |  |  |  |  |  |  |  | **Aminoglycoside resistance** | AAC(6')-Ic | 139431..139871 | aminoglycoside antibiotic |
|  |  |  |  |  |  |  |  |  |  | AAC(6')-Ib-cr6 | 244..843 | fluoroquinolone, ciprofloxacin, tobramycin, dibekacin, amikacin, netilmicin, sisomicin |
|  |  |  |  |  |  |  |  |  |  | AAC(3)-Ia | 83..616 | aminoglycoside antibiotic |
|  |  |  |  |  |  |  |  |  |  | ANT(3'')-IIa | 1650..2451 | aminoglycoside antibiotic |
|  |  |  |  |  |  |  |  |  |  | SRT-2 | 93059..94195 | cephalosporin |
|  |  |  |  |  |  |  |  |  | **Antibiotic target replacement protein** | dfrA1 | 119165..119638 | trimethoprim |
|  |  |  |  |  |  |  |  |  |  | sul1 | 1954..2820 | sulfonamide antibiotic |
|  |  |  |  |  |  |  |  |  | **Efflux pump** | acrB | 216255..219412 | antibiotic molecule |
|  |  |  |  |  |  |  |  |  |  | qacEdelta1 | 2787..3134 | benzylkonium chloride, ethidium bromide, chlorhexidine, cetylpyridinium chloride |
